# Enhanced Hygiene Measures and Norovirus Transmission during an Outbreak

**DOI:** 10.3201/1501.080299

**Published:** 2009-01

**Authors:** Janneke C.M. Heijne, Peter Teunis, Gabriella Morroy, Clementine Wijkmans, Sandy Oostveen, Erwin Duizer, Mirjam Kretzschmar, Jacco Wallinga

**Affiliations:** National Institute for Public Health and the Environment, Bilthoven, the Netherlands (J.C.M. Heijne, P. Teunis, E. Duizer, M. Kretzchmar, J. Wallinga); Emory University, Atlanta, Georgia, USA (P. Teunis); Municipal Health Services **“**Hart voor Brabant,” ‘s-Hertogenbosch, the Netherlands (G. Morroy, C. Wijkmans, S. Oostveen); University Medical Center Utrecht, Utrecht, the Netherlands (M. Kretzschmar, J. Wallinga); 1 These authors contributed equally to this article.

**Keywords:** Norovirus, outbreaks, hygiene measures, transmission, reproduction number, generation time distribution, research

## Abstract

Control of norovirus outbreaks relies on enhanced hygiene measures, such as handwashing, surface cleaning, using disposable paper towels, and using separate toilets for sick and well persons. However, little is known about their effectiveness in limiting further spread of norovirus infections. We analyzed norovirus outbreaks in 7 camps at an international scouting jamboree in the Netherlands during 2004. Implementation of hygiene measures coincided with an 84.8% (95% predictive interval 81.2%–86.6%) reduction in reproduction number. This reduction was unexpectedly large but still below the reduction needed to contain a norovirus outbreak. Even more stringent control measures are required to break the chain of transmission of norovirus.

## Abstract 2

Enhanced hygiene measures can reduce norovirus transmission potential by 85%.

Gastroenteritis is one of the most common causes of illness (*1*). Recent findings indicate norovirus is the most common cause of gastroenteritis (*2*,*3*). Of all gastroenteritis outbreaks reported in the Netherlands during 2002, 54% were caused by norovirus (*4*). Norovirus is predominantly transmitted through the fecal–oral route, either indirectly through contaminated food or surfaces or directly from person to person (*5*). It can be transmitted through small infectious droplets (aerosols) after a vomiting episode (*6*,*7*) and can survive for a very long time in the environment (*5*,*8*). Most norovirus outbreaks are seen in settings where clusters of vulnerable, susceptible persons live closely together, such as nursing homes, hospitals, and daycare centers (*4*), and in settings in which turnover of susceptible persons is high, such as hotels and cruise ships (*9*,*10*).

Norovirus infection can cause serious medical complications, such as dehydration, in persons with underlying illness (*11*). No antiviral treatment exists for norovirus infection, and although norovirus vaccines are in development (*12*), none are available yet. Early studies suggested that norovirus outbreaks could be contained by rapid implementation of enhanced hygiene measures, such as washing hands, thoroughly cleaning contaminated surfaces, avoiding contact between sick and healthy persons, and requesting caretakers and cleaning staff to wear gloves and aprons (*13*–*16*). However, in nursing homes or on cruise ships, norovirus can cause consecutive outbreaks, even after implementation of strict hygiene protocols (*9*,*17*,*18*). No quantitative estimates exist of the results of such enhanced hygiene measures on reducing further transmission of norovirus. To our knowledge, the effect of enhanced hygiene measures has not been investigated in randomized controlled trials or in statistical analyses of outbreaks.

We investigated the effect of enhanced hygiene measures on reducing norovirus transmission during an outbreak. We measured the effectiveness of enhanced hygiene measures as the relative reduction in the reproduction number—defined as the average number of secondary cases caused by 1 typical case—in the absence of and after enhanced hygiene measures. The value of this reproduction number provides crucial information about transmission potential: if the reproduction number exceeds the threshold value of 1, the number of new cases will increase over time; if it is <1, the number of new cases will decline over time, and eventually the chain of transmission will break.

The time course of the reproduction number during an outbreak can be inferred from the epidemic curve (*19*,*20*). We obtained a detailed epidemic curve of a norovirus outbreak at an international scout jamboree in the Netherlands from July 26 through August 10, 2004. This outbreak was ideally suited to estimating the effects of enhanced hygiene measures because the date enhanced hygiene measures began was recorded. Moreover, because the scouts were divided into 7 camps, the jamboree provided a natural experiment in which the camps could be regarded as “experimental units,” with varying durations between introduction of the virus and implementation of enhanced hygiene measures.

## Methods

### Data

An outbreak of norovirus infection occurred at an international scout jamboree in the Netherlands during the summer of 2004 (*21*). Approximately 4,500 persons from 32 countries attended this event. At the start of the scout jamboree on July 26, 2004, two participants became ill with symptoms of gastroenteritis. The outbreak affected at least 326 persons with typical, generally mild symptoms of gastroenteritis (case-patients). Most ill persons experienced vomiting (258) and/or diarrhea (195). Ninety-two ill persons visited a local first aid tent; another 54 were admitted to a local hospital for rehydration.

**Figure 1:**
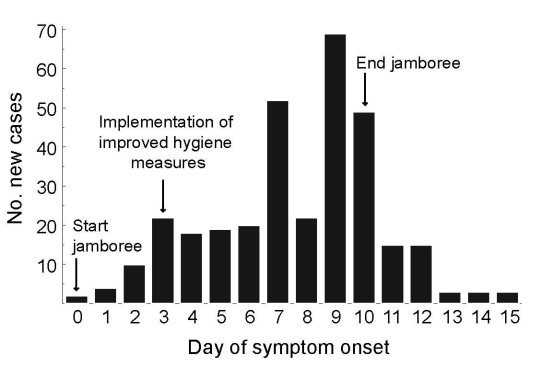
Epidemic curve of an outbreak of norovirus at an international scout jamboree in the Netherlands, starting July 26, 2004 (day 0).

The jamboree was held on a large site, ≈600 m × 1,000 m. Jamboree participants were divided into 7 camps according to age: 3 camps each for participants 11–14 and 15–17 years of age and 1 camp for staff >18 years of age. The 7 camps were situated around a central field for joint activities; most activities were organized within the camps. The camps were labeled A–G, according to the day the first participants became sick. For 296 (91%) of 326 case-patients, the camp label was known (Figure 1, Table).

**Table:** New norovirus cases during outbreak at international scout jamboree, the Netherlands, starting on Jul 26, 2004 (day 0), by day of symptom onset and camp label

Day of onset	Camp, no. new cases/d								Total (n = 4,500)
	A (n = 485)	B (n = 721)	C (n = 729)	D (n = 499)	E (n = 735)	F (n = 825*)	G (n = 506)	Unknown	
0	1	1	0	0	0	0	0	0	2
1	1	0	1	0	0	0	0	2	4
2	0	2	2	1	1	3	0	1	10
3	2	7	9	0	2	1	0	1	22
4	3	4	2	1	2	2	4	0	18
5	0	10	1	2	1	1	2	2	19
6	0	12	3	2	2	0	0	1	20
7	2	19	14	2	3	3	6	3	52
8	3	5	8	2	2	1	1	0	22
9	7	10	14	0	2	10	24	2	69
10	5	4	3	2	0	16	8	11	49
11	3	2	4	1	0	1	1	3	15
12	4	1	4	2	1	1	0	2	15
13	0	0	1	0	0	0	1	1	3
14	0	0	1	0	0	1	0	1	3
15	0	0	1	0	1	1	0	0	3
Total†	31	77	68	15	17	41	47	30	326

*This number is estimated. †Overall attack rate: 7.2. Attack rate by camp: A, 6.4; B, 10.7; C, 9.3; D, 3.0; E, 2.3; F, 5.0; G, 9.3.

On July 29 (day 3 of the jamboree), the Municipal Health Service “Hart voor Brabant” in ’s-Hertogenbosch provided advice on enhanced hygiene measures (*22*), instructed participants about proper hand hygiene and use of soap pumps and disposable paper towels, and assigned separate toilets for sick participants. In addition, the Municipal Health Service provided guidelines for cleaning toilets and contaminated surfaces with a 1,000-ppm chlorine solution. Sick participants were instructed to go to a first aid tent. Sick participants were not allowed to prepare food until 3 days after their last symptoms. Persons working in the jamboree’s field hospital were instructed to wear gloves, aprons, and surgical masks and to minimize the number of patients per nurse. The scout jamboree ended on August 5.

Norovirus was epidemiologically implicated as the causative agent (*21*) of the outbreak and was confirmed in stool samples through a standard reverse transcription-PCR protocol (*23*). Typing of 7 samples from case-patients in whom symptoms first developed 7–9 days after the outbreak began resulted in 3 norovirus genotypes: 2 samples typed as norovirus genotype GI.4, 1 sample typed as genotype GI.5, and 4 samples typed as genotype GII.4–2004. We did not detect any multiple infections.

During the outbreak, the Municipal Health Service assessed the number of new cases from typical gastroenteritis symptoms self-reported by participants and staff. After the jamboree, participants and staff were given a questionnaire asking them to report to the Municipal Health Service whether gastroenteritis had developed within a week after departure. The questionnaire asked the date of symptom onset, symptoms, camp label, and hospital admission.

### Reproduction Number

#### Estimation of Reproduction Numbers

**Figure 2:**
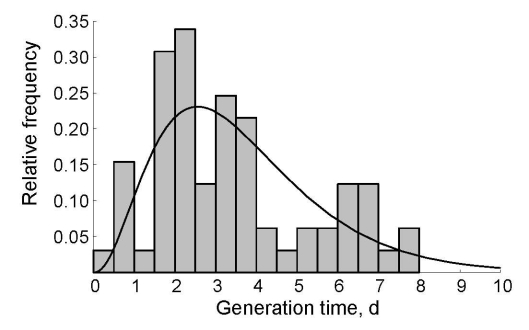
Generation time distribution for norovirus infections. Generation time is the time between onset of symptoms in successive case-patients. The histogram gives the relative frequency in norovirus outbreaks in Sweden in 1999 (*25*); the black line indicates the maximum-likelihood fit of the gamma distribution.

We estimated the reproduction number for every case during the norovirus outbreak at the jamboree. Using the date of symptom onset for each case, we applied statistical methods to reconstruct likely patterns of who infected whom (Technical Appendix 1). We first calculated the difference in day of symptom onset for all combinations of case pairs. To calculate the probability of transmission between any pair of cases, we needed information from the distribution of generation times (defined as the time between day of symptom onset in a case and day of onset in its primary case) (*19*,*24*). To estimate the generation time distribution for norovirus infections, we used observations of generation times from several large norovirus outbreaks in child daycare centers in Sweden during 1999 (*25*). These generation times were well described by a gamma distribution (Figure 2), for which the parameters were estimated by the method of maximum likelihood (Technical Appendix 1). The frequency distribution of generation times was used to assign a likelihood of transmission for any pair of cases, allowing estimation of the transmission probabilities. We then used a powerful statistical sampling algorithm to generate a large sample of plausible transmission patterns (for details, see Technical Appendix 1). The expected value of the reproduction number for a specific case was the sum of all transmission probabilities of outgoing infectious contacts to all other cases in the outbreak. For case in which symptoms began the same day, we calculated the mean minimum and maximum values of the reproduction number. For the entire sample of transmission probabilities, we obtained the 0.025 and 0.975 quantiles for these 3 metrics as predictive intervals.

##### Host Population Structure and Pathogen Genotype

We incorporated additional information about the camp label of almost all case-patients and the pathogen genotype for 7 case-patients into the estimation procedure by adding a ”weight” to the transmission probabilities between pairs of cases. Here we considered 2 extreme cases for mixing between camps. The first extreme case was homogeneous mixing between all participants of the jamboree, as we assumed in the analysis described above; to achieve this, we assigned a weight of 1 to any pair of cases. The second extreme case was mixing within camps only and no mixing between camps. In this instance, the transmission probabilities for pairs of case-patients that stayed in different camps were assigned a weight of 0, and the transmission probabilities for pairs of case-patients that stayed in the same camp were given a weight of 1. The transmission probabilities for pairs of cases with known different genotypes were assigned a weight of 0, and the transmission probabilities for pairs of cases with known identical genotypes were assigned a weight of 1.

##### Expected Time Course of Reproduction Number

If the enhanced hygiene measures resulted in a sudden decline in transmission, the expected decline of the reproduction number would be gradual. Four factors determined the expected time course: the day enhanced hygiene measures began, the cumulative frequency distribution of generation times, the reproduction number without enhanced hygiene measures, and the relative reduction of the reproduction number attributed to hygiene measures. We express the reproduction number as a function of these 4 factors (Technical Appendix 1) and fitted this function to every sampled time course of the mean reproduction number for days 0–5, with least squares regression to obtain point estimates and 95% predictive intervals for the parameters describing the reproduction number in the absence of hygiene measures and relative reduction of the reproduction number resulting from the hygiene measures.

##### Testing of the Estimation Procedures

We tested the estimation procedure by simulating epidemic curves with known reproduction numbers. The interval estimates for reproduction numbers covered the actual values for days 0–7. We detected a slight downward bias for the estimated value of reproduction numbers and a slight downward bias for the estimated relative reduction of reproduction numbers after implementation of enhanced hygiene measures, indicating that the values obtained by the estimation procedure are conservative (Technical Appendix 2).

## Results

**Figure 3:**
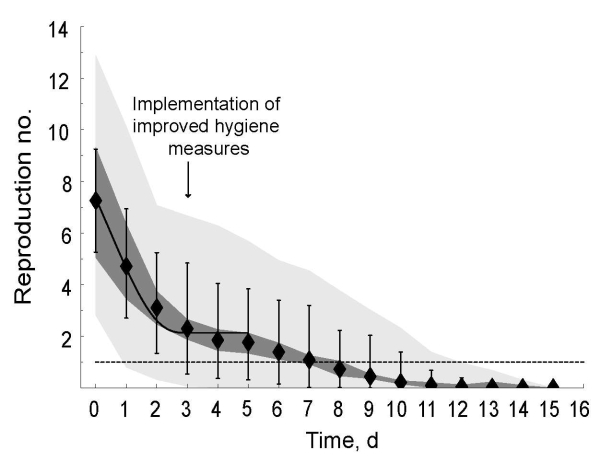
Time course of the reproduction number for norovirus at an international scout jamboree, starting July 26, 2004 (day 0), in the Netherlands. Black diamonds show the mean value for the reproduction number over all sampled transmission matrices; vertical lines, mean minimum and maximum values for the reproduction number over all sampled transmission matrices. The dark gray area shows the uncertainty range (0.025 and 0.975 quantiles) in the mean reproduction number; light gray are, the uncertainty range (0.025 and 0.975 quantiles) of the maximum and minimum estimates of the reproduction number. The solid black line represents the fitted time course of reproduction numbers if decrease in the mean reproduction number results from an instantaneous decline in transmission when enhanced hygiene measures began; dashed line, the threshold value of 1, below which the outbreak was controlled.

The estimated reproduction numbers decreased over time as the norovirus outbreak spread through the international scout jamboree (Figure 3). We estimated an initial reproduction number of 7.26 secondary cases per primary case (95% predictive interval 5.26–9.25); 5 days after the enhanced hygiene protocol began, the estimated reproduction number dropped below 1 (Figure 3, black diamonds). Under the hypothesis that transmission potential decreased abruptly when enhanced hygiene measures began, we estimated a reproduction number of 14.05 secondary cases per primary case (95% predictive interval 9.96–17.98) without enhanced hygiene measures and a reproduction number of 2.13 secondary cases per primary case (95% predictive interval 1.88–2.40) with enhanced hygiene measures (Figure 3, black solid line). This decrease corresponded to a relative reduction in reproduction number of 84.8% (95% predictive interval 81.2%–86.6%).

**Figure 4:**
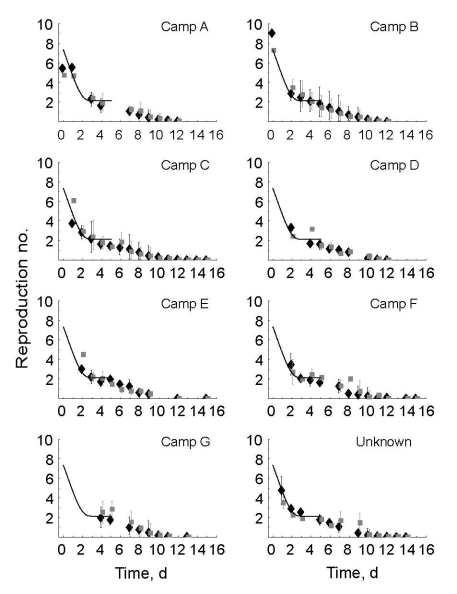
Time course of the reproduction number for norovirus for 7 camps of an international scout jamboree. Black diamonds show the mean value of the reproduction number without additional information about population structure and genotypes. Gray boxes show the mean value of the reproduction number when additional information about population structure and genotypes is used. The vertical lines show the mean minimum and maximum reproduction number over all sampled transmission matrices. The solid black line represents the time course of reproduction numbers if decrease in the mean reproduction number results from an instantaneous decline in transmission when enhanced hygiene measures begin. The camps are in order of the day of symptom onset of the first case-patient. Top panels indicate first introduction, bottom panels the last introduction.

The disease attack rate varied among different camps, from 2.3% to 10.7%; overall attack rate was 7.2% (Table). For camps A and B, the estimated time course of reproduction number was initially high for the 2 index case-patients (Figure 4, black diamonds). Repeating the analysis with additional information about the host population structure and pathogen genotypes resulted in similar point estimates of the reproduction numbers (Figure 4, gray boxes) but with narrower predictive intervals. The value of the initial reproduction number in each camp followed a time course consistent with 85% reduced transmission when enhanced hygiene measures were implemented (Figure 4, black solid lines), indicating the time course of the reproduction numbers did not depend on the time of introduction of norovirus in the camp—because this differed between camps—but on the time the enhanced hygiene protocol began, which was identical for all camps.

## Discussion

We have shown that during an outbreak of norovirus, implementation of enhanced hygiene measures coincided with an 85% reduction of norovirus transmission, from 14.05 secondary cases per primary case before enhanced hygiene measures to 2.13 secondary cases per primary case after enhanced hygiene measures. This estimate is consistent with the time course of reproduction numbers in different camps in which infection was introduced at different times. Our estimates confirm the alleged high epidemic potential of norovirus and suggest that the enhanced hygiene measures were not sufficient to reduce the reproduction number below the threshold value of 1. This estimate explains why the number of new cases per day continued to increase and why norovirus infection spread to new camps, even after implementation of enhanced hygiene measures. It is tempting to speculate that our findings could be extrapolated to other hygiene measures to explain the typical pattern in several subsequent norovirus outbreaks on cruise ships and in hotels (*9*,*26*,*27*).

The estimation procedure for the time course of the reproduction number has several limitations. It requires a frequency distribution for the generation time, which may be unknown for many diseases that are less well studied than norovirus. The procedure also requires reporting of symptom onset of case-patients over intervals on the order of the mean generation time or smaller. Here, because the mean generation time was 3.6 days, we cannot use weekly reports of time of symptom onset. The procedure also requires a large outbreak so the effects of chance events on the course of the epidemic are minimized. Small outbreaks would lead to estimates of reproduction numbers that are highly uncertain and have questionable value for making generalizations about transmission.

Our main result is that the observed decline in the reproduction number coincided with implementation of enhanced hygiene measures. This extrapolation is highly suggestive of a causal relationship, which implies that hygiene measures can effectively reduce transmission of norovirus. However, several alternatives can explain the declining reproduction number, as discussed below.

First, the decrease in reproduction number may be due to chance events. Here we explicitly estimated the reproduction numbers from times of symptom onset and the generation time distribution for norovirus infections, whereas earlier work relied on transforming epidemic curves to reproduction numbers (*19*,*20*). The tests of our explicit estimation procedure indicate that the interval estimates cover the actual values of reproduction numbers and the reduced reproduction numbers after the implementation of hygiene measures. The predictive interval for the relative reduction of 81.2%–86.6% clearly shows the change is statistically significant because it excludes the null hypothesis of a change of 0%. The tests also indicate a slight bias in the estimated values toward lower values, which suggests that the estimated 85% reduction after enhanced hygiene measures began should be treated as a conservative estimate. Therefore, the reduction in transmission is highly unlikely to be due to chance.

Second, it might be that jamboree participants differed in susceptibility, and the pool of highly susceptible persons was depleted during the first days of the outbreak. However, preexisting immunity for the genotypes involved seems highly unlikely: GI.5 and GI.4 rarely are detected in Europe, and the GII.4–2004 genotype caused a large epidemic during the winter after the jamboree (*28*). The number of persons infected before implementation of enhanced hygiene measures was smaller than the total number of case-patients, and the total number of case-patientss was smaller than the number of jamboree participants. Depletion of susceptible persons or different susceptibility is highly unlikely to explain the sudden decrease in transmission around day 3 of the outbreak.

Third, the decline in reproduction number could be because many infections were asymptomatic and many symptomatic cases were not reported. The request to report any symptoms might not have reached all participants because of the event’s large size and because participants came from many different countries. During norovirus outbreaks, asymptomatic cases occur; in almost half of the outbreaks in the Netherlands during 2002, stool samples from >1 healthy persons tested positive for norovirus (*4*). Volunteer and outbreak studies demonstrate that 30% of collected stool specimens of exposed, asymptomatic persons were positive for norovirus (*29*–*31*). However, both the proportion of asymptomatic infections and the reporting rate, as long as they remain constant, do not influence the value of the reproduction number because the reproduction number is estimated as the ratio of the number of secondary cases to the number of primary cases: both the proportion of asymptomatic infections and the reporting rate affect both the numerator and the denominator of this ratio, thereby canceling out in this calculation. Therefore, how the proportion of asymptomatic infections or the reporting rate would have resulted in a similar decline in reproduction number in the different camps is difficult to imagine.

Fourth, different genotypes of norovirus could have spread at different times during the outbreak. From genotyping data of 7 cases of the norovirus outbreak during the jamboree, we know that 3 different norovirus genotypes circulated during this outbreak from genogroup I and II. Recent work (*32*) showed first signs of a different viral load, which could indicate different transmissibility and different generation times between genogroup I and genogroup II. However, all genotyped strains were found during days 7–9 of the outbreak; although we cannot rule out the possibility that genotype replacement occurred, the most transmissible type is highly unlikely to have dominated during the first 3 days before giving way to less transmissible types.

Finally, a change of the generation time distribution during the outbreak could explain the decline in reproduction number. The method we used to estimate the time course of reproduction number depends crucially on a correct specification of the generation time distribution. Here we obtained this distribution from a study of a norovirus outbreak in child daycare centers in Sweden (*25*) and estimated that the generation time distribution peaked at 2.6 days (Figure 2). This estimation agrees with results from volunteer studies in which adults showed a peak in virus shedding at 1–3 days postchallenge (*31*). Further, this peak agrees with the time between exposure and symptom onset of 2 days in primary-school children during a norovirus outbreak after a vomiting event (*6*), whereas 80% of case-patients reported vomiting during the scout jamboree. Overall, the most plausible explanation for the decrease in reproduction number is implementation of enhanced hygiene measures.

We have quantitatively estimated the effectiveness of enhanced hygiene measures in containing an outbreak of norovirus. Because the reproduction number did not fall below the threshold value of 1, implementation of the hygiene measures was not sufficient to effectively break the chain of person-to-person transmission of norovirus during this outbreak. To contain an outbreak of norovirus, more rigorous interventions are required. These might range from better compliance with hygiene protocols to strict isolation of case-patients and quarantine of their contacts. We recommend quantifying the effectiveness of interventions against norovirus to provide the necessary evidence to justify use of existing hygiene protocols during outbreaks and to direct the development of better intervention measures. Although such quantifying would require analysis of more norovirus outbreaks with different sets of intervention measures, it would enable identification of the best possible intervention strategies to control the spread of one of the most common pathogens of humans.

## Supplementary Material

Technical Appendix 1 Methods

Technical Appendix 2 Testing the Estimation Procedures with Simulated Outbreaks

## References

[1] Mead PS, Slutsker L, Dietz V, McCaig LF, Bresee JS, Shapiro C, Food-related illness and death in the United States. Emerg Infect Dis. 1999;5:607–25. 10511517 10.3201/eid0505.990502 PMC2627714

[2] Estes MK, Prasad BV, Atmar RL. Noroviruses everywhere: has something changed? Curr Opin Infect Dis. 2006;19:467–74. 10.1097/01.qco.0000244053.69253.3d 16940871

[3] Kroneman A, Vennema H, Harris J, Reuter G, von Bonsdorff CH, Hedlund KO, Increase in norovirus activity reported in Europe. Euro Surveill 2006;11(50):3093 [cited 17 Nov 2008]. Available from http://www.eurosurveillance.org/ViewArticle.aspx?ArticleId=3093 10.2807/esw.11.50.03093-en 17213565

[4] van Duynhoven YT, de Jager CM, Kortbeek LM, Vennema H, Koopmans MP, van Leusden F, A one-year intensified study of outbreaks of gastroenteritis in the Netherlands. Epidemiol Infect. 2005;133:9–21. 10.1017/S0950268804002936 15724705 PMC2870216

[5] Duizer E, Koopmans M. Tracking foodborne viruses: lessons from noroviruses. In: Motarjemi Y, Adams M, editors. Emerging foodborne pathogens. Boca Raton (FL): CRC Press, 2006. p. 77–110.

[6] Evans MR, Meldrum R, Lane W, Gardner D, Ribeiro CD, Gallimore CI, An outbreak of viral gastroenteritis following environmental contamination at a concert hall. Epidemiol Infect. 2002;129:355–60. 10.1017/S0950268802007446 12403111 PMC2869894

[7] Marks PJ, Vipond IB, Carlisle D, Deakin D, Fey RE, Caul EO. Evidence for airborne transmission of Norwalk-like virus (NLV) in a hotel restaurant. Epidemiol Infect. 2000;124:481–7. 10.1017/S0950268899003805 10982072 PMC2810934

[8] Barker J, Vipond IB, Bloomfield SF. Effects of cleaning and disinfection in reducing the spread of norovirus contamination via environmental surfaces. J Hosp Infect. 2004;58:42–9. 10.1016/j.jhin.2004.04.021 15350713

[9] Isakbaeva ET, Widdowson MA, Beard RS, Bulens SN, Mullins J, Monroe SS, Norovirus transmission on cruise ship. Emerg Infect Dis. 2005;11:154–8. 15705344 10.3201/eid1101.040434 PMC3294347

[10] Love SS, Jiang X, Barrett E, Farkas T, Kelly S. A large hotel outbreak of Norwalk-like virus gastroenteritis among three groups of guests and hotel employees in Virginia. Epidemiol Infect. 2002;129:127–32. 10.1017/S0950268802007161 12211579 PMC2869857

[11] Rockx B, De Wit M, Vennema H, Vinje J, De Bruin E, Van Duynhoven Y, Natural history of human calicivirus infection: a prospective cohort study. Clin Infect Dis. 2002;35:246–53. 10.1086/341408 12115089

[12] LoBue AD, Lindesmith L, Yount B, Harrington PR, Thompson JM, Johnston RE, Multivalent norovirus vaccines induce strong mucosal and systemic blocking antibodies against multiple strains. Vaccine. 2006;24:5220–34. 10.1016/j.vaccine.2006.03.080 16650512

[13] Calderon-Margalit R, Sheffer R, Halperin T, Orr N, Cohen D, Shohat T. A large-scale gastroenteritis outbreak associated with norovirus in nursing homes. Epidemiol Infect. 2005;133:35–40. 10.1017/S0950268804003115 15724708 PMC2870219

[14] Cheng FW, Leung TF, Lai RW, Chan PK, Hon EK, Ng PC. Rapid control of norovirus gastroenteritis outbreak in an acute paediatric ward. Acta Paediatr. 2006;95:581–6. 10.1080/08035250500449874 16825139

[15] Navarro G, Sala RM, Segura F, Arias C, Anton E, Varela P, An outbreak of norovirus infection in a long-term-care unit in Spain. Infect Control Hosp Epidemiol. 2005;26:259–62. 10.1086/502536 15796277

[16] Schmid D, Lederer I, Pichler AM, Berghold C, Schreier E, Allerberger F. An outbreak of norovirus infection affecting an Austrian nursing home and a hospital. Wien Klin Wochenschr. 2005;117:802–8. 10.1007/s00508-005-0473-1 16437316

[17] Cheesbrough JS, Green J, Gallimore CI, Wright PA, Brown DW. Widespread environmental contamination with Norwalk-like viruses (NLV) detected in a prolonged hotel outbreak of gastroenteritis. Epidemiol Infect. 2000;125:93–8. 10.1017/S095026889900432X 11057964 PMC2869574

[18] Verhoef L, Depoortere E, Boxman I, Duizer E, Van Duynhoven Y, Harris J, Emergence of new norovirus variants on spring cruise ships and prediction of winter epidemics. Emerg Infect Dis. 2008;14:238–43. 10.3201/eid1402.061567 18258116 PMC2600213

[19] Wallinga J, Teunis P. Different epidemic curves for severe acute respiratory syndrome reveal similar impacts of control measures. Am J Epidemiol. 2004;160:509–16. 10.1093/aje/kwh255 15353409 PMC7110200

[20] Cauchemez S, Boelle PY, Donnelly CA, Ferguson NM, Thomas G, Leung GM, Real-time estimates in early detection of SARS. Emerg Infect Dis. 2006;12:110–3. 16494726 10.3201/eid1201.050593 PMC3293464

[21] Duizer E, Timen A, Morroy G, de Roda Husman AM. Norovirus outbreak at an international scout jamboree in the Netherlands, July–August 2004: international alert. Euro Surveill. 2004;8(33):2523 [cited 17 Nov 2008]. Available from http://www.eurosurveillance.org/ViewArticle.aspx?ArticleId=2523

[22] Morroy G, Wijkmans C. A norovirus outbreak at an international scouting jamboree in the Netherlands [in Dutch]. Infectieziekten bulletin. 2005;16:57–9 [cited 17 Nov 2008]. Available from http://www.rivm.nl/infectieziektenbulletin/bul1602/veld_jamboree.html

[23] Vennema H, De Bruin E, Koopmans M. Rational optimization of generic primers used for Norwalk-like virus detection by reverse transcriptase polymerase chain reaction. J Clin Virol. 2002;25:233–5. 10.1016/S1386-6532(02)00126-9 12367660

[24] Fine PE. The interval between successive cases of an infectious disease. Am J Epidemiol. 2003;158:1039–47. 10.1093/aje/kwg251 14630599

[25] Götz H, Ekdahl K, Lindback J, de Jong B, Hedlund KO, Giesecke J. Clinical spectrum and transmission characteristics of infection with Norwalk-like virus: findings from a large community outbreak in Sweden. Clin Infect Dis. 2001;33:622–8. 10.1086/322608 11477530

[26] Enserink M. Infectious diseases: gastrointestinal virus strikes European cruise ships. Science. 2006;313:747. 10.1126/science.313.5788.747a 16902100

[27] Koopmans M, Harris J, Verhoef L, Depoortere E, Takkinen J, Coulombier D. European investigation into recent norovirus outbreaks on cruise ships: update. Euro Surveill. 2006;11(27):2997 [cited 17 Nov 2008]. Available from http://www.eurosurveillance.org/ViewArticle.aspx?ArticleId=2997 10.2807/esw.11.27.02997-en 16966757

[28] Siebenga JJ, Vennema H, Renckens B, De Bruin E, van der Veer B, Siezen RJ, Epochal evolution of GGII.4 norovirus capsid proteins from 1995 to 2006. J Virol. 2007;81:9932–41. 10.1128/JVI.00674-07 17609280 PMC2045401

[29] Gallimore CI, Cubitt D, du Plessis N, Gray JJ. Asymptomatic and symptomatic excretion of noroviruses during a hospital outbreak of gastroenteritis. J Clin Microbiol. 2004;42:2271–4. 10.1128/JCM.42.5.2271-2274.2004 15131210 PMC404621

[30] Garcia C, DuPont HL, Long KZ, Santos JI, Ko G. Asymptomatic norovirus infection in Mexican children. J Clin Microbiol. 2006;44:2997–3000. 10.1128/JCM.00065-06 16891526 PMC1594604

[31] Graham DY, Jiang X, Tanaka T, Opekun AR, Madore HP, Estes MK. Norwalk virus infection of volunteers: new insights based on improved assays. J Infect Dis. 1994;170:34–43. 8014518 10.1093/infdis/170.1.34

[32] Chan MC, Sung JJ, Lam RK, Chan PK, Lee NL, Lai RW, Fecal viral load and norovirus-associated gastroenteritis. Emerg Infect Dis. 2006;12:1278–80. 16965715 10.3201/eid1208.060081 PMC3291221

