# Supplementary material for: Enhanced Hygiene Measures and Norovirus Transmission during an Outbreak
Source: Emerg Infect Dis. 2009 Jan;15(1):24–30. doi: 10.3201/1501.080299 (PMC2660689; doi:10.3201/1501.080299)
Supplement: Technical Appendix 1 — Methods [file 08-0299_Techapp1-s1.pdf]

Enhanced Hygiene Measures and Norovirus Transmission during an Outbreak

Technical Appendix 1: Methods

Estimation of the Generation Time Distribution

Time interval data from large norovirus outbreaks in Sweden in 1999 (norovirus genogroup II) in childcare centers (*1*) were used to estimate the generation time distribution by the maximum likelihood method (Figure 2 in main text). The input data consist of a vector of the observed time intervals **s**, with $s_1, s_2, \ldots s_n$ denoting the times between symptom onset in persons who attended a childcare center and the times of symptom onset in household members of the infected persons.

We assume that the generation time distribution follows a gamma distribution with a shape parameter α and a scale parameter β. The log-likelihood for the observed time intervals **s**, given the parameters for the generation time, is

$$l(\alpha, \beta \mid \mathbf{s}) = \log\left(\prod_{i=1}^{n} Gamma(s_i \mid \alpha, \beta)\right).$$

The estimated maximum likelihood estimates are $\alpha = 3.35$ and $\beta = 1.09$, resulting in a peak generation time of 2.6, and a mean generation time of 3.6 days. Other positively skewed unimodal distributions such as the Weibull distributions did not produce a significantly better fit. As the generation time distribution might also be a realization of a mixture of several components, we fitted the data with a mixture of 2 or 3 gamma distributed components. This did not give a significantly better fit than a 1-component model (Technical Appendix 1 Table).

Technical Appendix 1 Table. Summary of a fit of gamma distribution with 1, 2, or 3, components, respectively, to the serial interval data

No. components	*log* likelihood	Deviance	Degrees of freedom	p value
1	345.176			
2	338.290	6.886	3	0.076
3	332.609	12.567	6	0.051

Estimation of the Effective Reproduction Number, R

Definition of Transmission Matrix

Let $\mathbf{t} = (t_1,\ldots,t_n)$ be the vector of observed times of symptom onset of observed cases {1, …, n}. We assume that the elements of t are ordered such that $t_i \leq t_j$ for all $i < j$. For subsets $\{i_k, \ldots i_{k+j}\} \subseteq \{1,\ldots,n\}$ with $t_{i_k} = t_{i_{k+1}} = \ldots = t_{i_{k+j}}$ all permutations of observations within this subset are equivalent. We chose 1 possible ordering arbitrarily. We now define a transmission matrix $V = (v_{i,j})$, whose elements represent the probability that the person with time of symptom onset t_i was infected by the person with time of symptom onset t_j, thus $v_{i,j} \in [0,1]$. Assuming that every case $i \geq 2$ was infected by another case in the set of observed cases, we get:

$$\sum_{j=1}^{n} v_{i,j} = 1$$

for all $i \geq 2$. For $i = 1$, the index case, we assume that:

$$\sum_{j=1}^{n} v_{i,j} = 0\,.$$

Furthermore, we assume that $v_{i,j} = 0$ for all $j \leq i$. This assumption means that the ordering of times of infection is equivalent to the ordering of observed times of symptom onset, and more specifically, that persons cannot have infected themselves and cannot have infected persons with earlier time of symptom onset than their own. The matrix V is a lower triangular matrix and therefore does not contain cycles.

Translation of Transmission Matrix to Reproduction Number Estimates

To translate the transmission matrix V to reproduction number estimates, any transmission matrix V may represent many different transmission trees. A transmission tree consists of nodes representing all cases of the outbreak and direct edges between nodes representing transmission of infection between the cases.

Let a transmission tree be represented by a binary matrix $U = (u_{i,j})$ of infectious contacts with $u_{i,j} = 1$ if case i is infected by case j and $u_{i,j} = 0$ if case i is not infected by case j. The row vector in matrix **U** can be seen as a draw from a multinomial distribution of order 1 (each case-patient received his or her infection from exactly 1 other case-patient) and a probability equal to

a row from matrix V, producing a vector u_i of 0s and only 1 element equal to 1: u_i = *multinomial*(1, v_i).

With a transmission tree, it is possible to simulate an epidemic curve. For any pair of cases i, j of which $u_{i,j} = 1$ draw a generation time τ_i from the generation time distribution $g(\tau \mid \theta)$, with θ being the parameters of the generation time distribution. With the generation time τ_i, the time of infection of case i can be determined: $t_i = t_j + \tau_i$. If the time of infection is known from the index case, all times of infections in all other cases can be determined, which results in an epidemic curve.

The expected number of secondary cases produced by case j in these possible outbreaks based on transmission matrix V is:

$$\underset{v}{E}(R_j) = \sum_i E(u_{i,j}) = \sum_i v_{i,j}$$

To translate this to an estimate of R for each day in the outbreak t; the mean R_j of all cases with the same date of symptom onset is calculated, for all dates with observations:

$$\underset{v}{E}(R(t)) = \frac{1}{q} \sum_{j=m}^{m+q} \underset{v}{E}(R_j)$$

where m represents the label of the first case with symptom onset on day t, and q the total number of cases on day t.

Likelihood Function

The likelihood that an observed time interval $t_i - t_j$ represents a transmission event is determined as a product of the probability that i was infected by j and the probability that the time interval of symptom onset is $t_i - t_j$. That is,

$$L_{i,j}(\theta, v_{i,j} \mid t_i - t_j) = g(t_i - t_j \mid \theta) \cdot v_{i,j}$$

The likelihood of any case-patient j transmitting infection to case-patient i, becomes:

$$L_i(\theta, v_i \mid t_i - t_j) = \sum_{j=1}^{n} \left(g(t_i - t_j \mid \theta) \cdot v_{i,j} \right)$$

Combining for all observed cases, the likelihood of a transmission matrix **V** becomes:

$$L(\mathbf{V}|\mathbf{t}) = \prod_{i=2}^{n}\left(\sum_{j=1}^{n}\left(g(t_i - t_j \mid \theta)\cdot v_{i,j}\right)\right)$$

for a given value of θ, and omitting the index case ($i = 1$) from the multiplication. Given the parameters for the generation time distribution θ, and all dates of symptom onset t, the parameters $v_{i,j}$ can be estimated. To estimate $v_{i,j}$, the above likelihood function was evaluated in an adaptive rejection algorithm (Metropolis Hastings sampler) obtaining sets of V matrices with relative frequencies proportional to their likelihood (*2–4*).

To be reasonably certain of convergence and sufficient mixing, we have run 4 independent chains of 40,000 iterations and 3 independent chains with additional information about population structure and pathogen genotype and compared resulting estimates of reproduction numbers.

Adding additional information is possible by setting implausible transmission probabilities in the transmission matrix V to 0. This may be considered a very strong prior assumption, but we have seen (Figure 4 in main text) that the resulting reproduction numbers are not strongly influenced by this radical assumption. In a true Bayesian approach, we might have applied different weights to pairs of cases within and between camps by multiplying a matrix containing these weights with the transmission matrix V.

As described above, case-patients with a date of symptom onset on the same day are given an arbitrary order of infection within that day. Sampled transmission matrices represent all possible (noncyclic) patterns among cases, given the arbitrary order. Now any other possible pattern can be found by permutation of indexes among cases with the same date of symptom onset. Because these all have the same contribution to the likelihood such permutations do not change the likelihood: all permutations are equally likely. Such permutations also have the same reproduction numbers, only for different cases (indices). If we average over all such permutations with identical contributions, the resulting reproduction numbers do not change.

Expected Time Course of Reproduction Number

The expected time course of the reproduction number $R(t)$ is given by the following equation:

$$R(t) = \left(G(t_h - t) + \left(1 - G(t_h - t)\right)\rho\right)R_u$$

Here, t_h is the day of implementation of enhanced hygiene measures, G is the cumulative probability function of the generation time distribution, ρ is the relative reduction of the reproduction number due to implementation of hygiene measures and R_u is the effective reproduction number without enhanced hygiene measures.

References

1. Götz H, Ekdahl K, Lindback J, de Jong B, Hedlund KO, Giesecke J. Clinical spectrum and transmission characteristics of infection with Norwalk-like virus: findings from a large community outbreak in Sweden. Clin Infect Dis. 2001;33:622–8. PubMed DOI: 10.1086/322608

2. Gilks WR, Richardson S, Spiegelhalter DJ. Markov chain Monte Carlo in practice. London: Chapman & Hall/CRC Press; 1995.

3. O'Neill PD. A tutorial introduction to Bayesian inference for stochastic epidemic models using Markov chain Monte Carlo methods. Math Biosci. 2002;180:103–14. PubMed DOI: 10.1016/S0025-5564(02)00109-8

4. O'Neill PD, Marks PJ. Bayesian model choice and infection route modelling in an outbreak of Norovirus. Stat Med. 2005;24:2011–24. PubMed DOI: 10.1002/sim.2090
