# Supplementary material for: Enhanced Hygiene Measures and Norovirus Transmission during an Outbreak
Source: Emerg Infect Dis. 2009 Jan;15(1):24–30. doi: 10.3201/1501.080299 (PMC2660689; doi:10.3201/1501.080299)
Supplement: Technical Appendix 2 — Testing the Estimation Procedures with Simulated Outbreaks [file 08-0299_Techapp2-s2.pdf]

Enhanced Hygiene Measures and Norovirus Transmission during an Outbreak

Technical Appendix 2: Testing the Estimation Procedures with Simulated Outbreaks

Simulation Study 1: Testing the Estimated Time Course of Reproduction Numbers

We used an individual-based stochastic model to simulate 50 epidemic curves. For any case with symptom onset at day t, the number of secondary cases is sampled from a geometric distribution with a mean equal to $R(t)$ as estimated from the outbreak data (black diamonds in Figure 3 in the main article). For each of these secondary cases, the generation time is sampled from a gamma distribution with parameters $\alpha = 3.35$ and $\beta = 1.09$ with a mean of 3.6 days, as estimated from the observed generation times (Technical Appendix 1, available from www.cdc.gov/EID/content/15/1/24-Techapp1.pdf). Each simulated outbreak started with 3 initial cases at day 0.

We used the same estimation procedure as described in Technical Appendix 1 to estimate the time course of the mean value of the reproduction numbers $R(t)$. We used fewer samples of the transmission matrix than for the actual estimates in the main text.

Simulation Study 2: Testing Estimation of Impact of Intervention Measures

We simulated again 50 epidemic curves with an individual-based stochastic model. For each case, the number of secondary cases is sampled from a geometric distribution with mean corresponding to the estimated mean reproduction number without enhanced hygiene measures R_u of 14.05, and an instantaneous decrease in reproduction number ρ of 85% when enhanced hygiene measures are implemented (black solid line in Figure 3 in main text).

Evaluation of Simulation Studies

The test results show that the point estimates of reproduction numbers in simulated outbreaks closely follows the actual value of reproduction numbers, but are biased toward lower values than the actual ones (Technical Appendix 2 Table 1). The ranges of estimated reproduction numbers cover the actual values. The test results also show a downward bias in the estimates of the reproduction number without enhanced hygiene measures R_u and the relative reduction in reproduction numbers ρ (Technical Appendix 2 Table 2). The downward bias can be attributed to the so-called attenuation bias of the least squares regression that was used to estimate the parameters R_u and ρ. Attenuation bias is caused by random noise in the explanatory variable, which induces a bias in the estimated regression coefficient toward 0. Here, random noise is introduced in the time of symptom onset by the variability in generation times, and this causes a bias of the parameter ρ toward 0.

Technical Appendix 2 Table 1. Test results for the estimation procedure of reproduction numbers*

Parameter	Actual value	Estimated value, mean (range)
$R(0)$	7.3	5.1(1.7–8.3)
$R(1)$	4.7	3.4(1.6–4.8)
$R(2)$	3.1	2.7(0.6–3.6)
$R(3)$	2.3	2.1(0.8–3.0)
$R(4)$	1.9	1.8(0.6–2.3)
$R(5)$	1.8	1.4(0.2–1.8)
$R(6)$	1.4	1.1(0.04–1.5)
$R(7)$	1.1	0.8(0.3–1.2)

*Range, minimum and maximum value of the estimated mean reproduction numbers in 50 simulations; $R(t)$, mean reproduction number of cases with symptom onset on day t in 50 simulations.

Technical Appendix 2 Table 2. Test results for the estimation procedure of the impact of enhanced hygiene measures*

Parameter	Actual value	Estimated value, mean (range)
R_u	14.1	9.5(3.7–15.2)
$(1-\rho)R_u$	2.1	2.1(1.2–2.6)
ρ	0.85	0.77(0.59–0.86)

*Range, minimum and maximum value of the estimated mean parameters in 50 simulations; R_u, mean reproduction number without enhanced hygiene measures; $(1-\rho)R_u$, mean reproduction number with enhanced hygiene measures; ρ, relative reduction in reproduction number when enhanced hygiene measures began.
